# Autologous or allogeneic hematopoietic stem cells transplantation combined with high-dose chemotherapy for refractory neuroblastoma: A protocol for systematic review and meta-analysis

**DOI:** 10.1097/MD.0000000000028096

**Published:** 2021-12-10

**Authors:** Zhang-Shuai Zhao, Wei Shao, Ji-Ke Liu

**Affiliations:** Department of Pediatric Surgery, Liaocheng People's Hospital, Shandong, PR China.

**Keywords:** efficacy, hematopoietic stem cells, meta-analysis, refractory neuroblastoma, survival

## Abstract

**Background:**

Neuroblastoma is a common solid malignant tumor in children. Despite the development of new treatment options, the prognosis of high-risk neuroblastoma patients is still poor. High-dose chemotherapy and hematopoietic stem cell (HSC) transplantation might improve survival of patients with refractory neuroblastoma. In this study, we aimed to summarize the efficacy of autologous or allogeneic HSC transplantation combined with high-dose chemotherapy for patients with refractory neuroblastoma through the meta-analysis.

**Methods and analysis:**

Relevant clinical trials of autologous or allogeneic HSC transplantation for the treatment refractory neuroblastoma patients will be searched in Web of Science, Cochrane Library, PubMed, Google Scholar, Embase, Medline, China National Knowledge Infrastructure, China Scientific Journal Database, Chinese Biomedical Literature Database and Wanfang Database from their inception to December 2020. Two researchers will perform data extraction and risk of bias assessment independently. The clinical outcomes including tumor response, overall survival, event-free survival (EFS), quality of life (QoL) and adverse events, were systematically evaluated by using Review Manager 5.3 and Stata 14.0 statistical software.

**Results:**

The results of this study will provide high-quality evidence for the effect of autologous or allogeneic HSC transplantation combined with high-dose chemotherapy on tumor response, survival, and QoL in patients with refractory neuroblastoma.

**Conclusions:**

The conclusions of this meta-analysis will be published in a peer-reviewed journal, and provide more evidence-based guidance in clinical practice.

## 1 Introduction

Neuroblastoma is the second most common solid malignant tumor in children, only next to central nervous system tumors.^[1–3]^ It arises from the developing sympathetic nervous system from neural crest cells, usually resulting in tumors in the adrenal glands or the sympathetic ganglia.^[1,4,5]^ The age-standardized annual incidence in North America is 5.5 to 11.5 cases per million people.^[1,6]^ It is the most common malignancy overall in the first year of life with a median age at diagnosis of 18 months and 90% of cases diagnosed by 10 years of age.^[1,7–9]^ Conventional treatment options for refractory neuroblastoma include surgery, radiotherapy, chemotherapy, immunotherapy, autologous stem cell transplant, or a combination of them, depending on the severity of the disease.^[9–14]^ With the improvement of therapeutic methods, although the 5-year survival rate of patients with neuroblastoma has increased from 29% to 50% over the past 2 decades, the long-term outcome of refractory neuroblastoma remains unsatisfactory.^[1,8,10]^ Some progress in the treatment of high-risk neuroblastoma is closely related to the escalation of therapeutic intensity.^[1,14,15]^ However, high-dose chemotherapy can also seriously damage the hematopoietic system of patients.^[14,15]^

**Figure 1:**
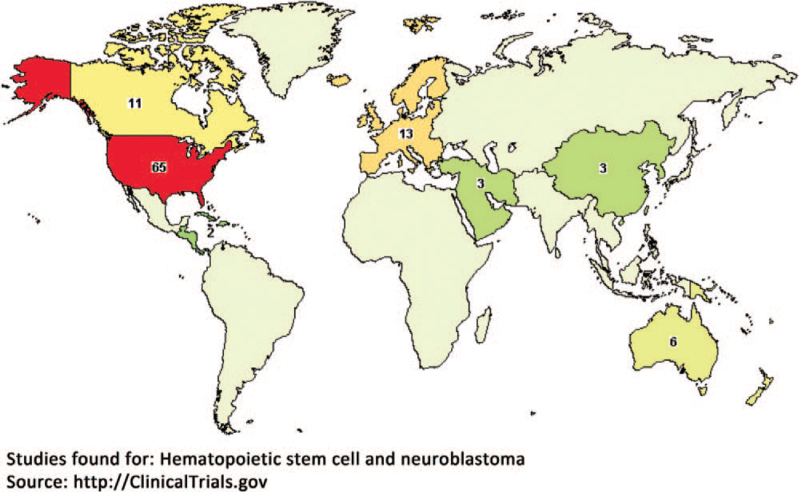
Clinical trials registration map.

Hematopoietic stem cell (HSC) is a kind of stem cell in bone marrow, peripheral blood or cord blood.^[16,17]^ It has the ability of self-renewal and can differentiate into a variety of blood cell precursor cells, and finally generate various blood cell components, including red blood cells, white blood cells and platelets.^[16–19]^ Healthy HSC are capable of long-term multilineage reconstitution and in situ recovery of the hematopoietic system (e.g., after massive cytotoxic injury induced by radiation or chemotherapy).^[19]^ In order to achieve dose escalation beyond marrow tolerance, HSC transplantation has been used for adjuvant high-dose chemotherapy against refractory neuroblastoma.^[14,15,20–25]^ Currently, a great deal of clinical trials in which neuroblastoma is being treated by high-dose chemotherapy in conjunction with HSC transplantation have been registered on ClinicalTrials.gov (Fig. 1). Several studies have indicated that the combination of autologous or allogeneic HSC transplantation and high-dose chemotherapy not only exerts an enhanced therapeutic effect against refractory neuroblastoma, but also improve the quality of life (QoL) patients.^[20–26]^ Despite the intensive clinical studies, its clinical efficacy was still not well investigated. In this study, we are prepared to summarize the efficacy of autologous or allogeneic HSC transplantation on tumor response, survival and QoL in patients with refractory neuroblastoma through the meta-analysis, in order to provide a helpful evidence for clinicians to formulate the best treatment strategy for refractory neuroblastoma patients.

## 2 Review question

Is autologous or allogeneic HSC transplantation effective on tumor response, survival, and QoL in patients with refractory neuroblastoma?

## 3 Objective

A systematic review and meta-analysis will be performed to systematically evaluate the efficacy of autologous or allogeneic HSC transplantation on tumor response, survival, QoL and adverse effect in patients with refractory neuroblastoma.

## 4 Methods and analysis

### 4.1 Study registration

The protocol has been registered on the International Platform of Registered Systematic Review and Meta-Analysis Protocols. The registration number was INPLASY2021110007 (URL: https://inplasy.com/inplasy-2021–11–0007/). This protocol of systematic review and meta-analysis will be reported according to Preferred Reporting Items for Systematic Review and Meta-Analysis Protocols (PRISMA-P) guidelines.^[27]^

### 4.2 Ethics

No further ethical approval is required since the program does not require the recruitment of patients and the collection of personal information.

### 4.3 Eligibility criteria

#### 4.3.1 Types of studies

Randomized controlled trials (RCTs) or prospective controlled clinical trials that investigated the efficacy and safety of autologous or allogeneic HSC transplantation for patients diagnosed with refractory neuroblastoma will be included in this systematic review. There will be no restrictions for blinding, population characteristics and duration of trials.

#### 4.3.2 Type of participants

Patients with histologically proved refractory neuroblastoma (High risk according Children Oncology Group or Refractory) were included in this study. No restrictions regarding age, gender, racial, region, education and economic status. Patients with other malignancies are not included.

#### 4.3.3 Types of interventions

In the experimental group, refractory neuroblastoma patients must be treated with autologous or allogeneic HSC transplantation in combination with high-dose chemotherapy. There will be no restrictions with respect to dosage, duration, frequency, or follow-up time of treatment.

#### 4.3.4 Comparator

In the control group, patients with refractory neuroblastoma must be treated with high-dose chemotherapy.

### 4.4 Type of outcome measurements

#### 4.4.1 Primary outcomes

Tumor response (complete response, very good partial response, and partial response). It will be assessed on day 60 after HSC transplantation. Such evaluations will include 123I-MIBG scan, CT/MRI, and urine catecholamine measurement, et al;

1. Overall survival (OS, from 1-, 3-, and 5-year after HSC transplantation), It will be measured from the date of randomization to death from any cause;
2. Event-free survival (EFS, from 1-, 3-, and 5-year after HSC transplantation). It will be measured from start of treatment until progression, death or start of another treatment.

#### 4.4.2 Secondary outcomes

QoL obtained from the corresponding scale;

1. Safety assessment. Monitoring of mortality, toxicity (NCI Common Criteria), acute and chronic graft versus host disease, and engraftment rate will contribute to safety assessment.
2. **Exclusion criteria.** Duplicated studies, non-comparative clinical trials, papers without sufficient available data, meta-analysis, literature reviews, meeting abstracts, case reports and series, and other unrelated studies will be excluded from analysis.

### 4.5 Information sources

Relevant clinical trials of autologous or allogeneic HSC transplantation for the treatment refractory neuroblastoma patients will be searched in Web of Science, Cochrane Library, PubMed, Google Scholar, Embase, Medline, China National Knowledge Infrastructure, China Scientific Journal Database, Chinese Biomedical Literature Database and Wanfang Database from their inception to December 2020. Language is limited with English and Chinese.

### 4.6 Search strategy

Experienced systematic review investigators will be invited to develop a search strategy, in order to perform a comprehensive search. The search terms include “neuroblastoma” or “refractory neuroblastoma” or “high-risk neuroblastoma” and “stem cell” or “stem cell transplantation” or “hematopoietic stem cell” or “hematopoietic stem cell transplantation” or “autologous hematopoietic stem cell transplantation” or “allogeneic hematopoietic stem cell transplantation” or “HSC” and “Chemotherapy” or “High-dose chemotherapy” et al. The preliminary retrieval strategy for PubMed is provided in Table 1, which will be adjusted in accordance with specific databases.

**Table 1:** Searching strategy in PubMed.

Search strategy
#1. “Neuroblastoma” or “Neuroblastomas” or “Ganglioneuroblastoma” or “Ganglioneuroblastomas” or “Refractory neuroblastoma” or “High-risk neuroblastoma” [Title/Abstract].
#2. “Neuroblastoma” [MeSH].
#3. #1 or #2.
#4. “Stem cell” or “Stem cell transplantation” or “Stem cell rescue” or “Bone marrow transplantation” or “Bone marrow grafting” or “Peripheral blood stem cell transplantation” or “Umbilical cord stem cell transplantation” or “Bone marrow grafting” or “Hematopoietic stem cell” or “Hematopoietic stem cell transplantation” or “Autologous hematopoietic stem cell transplantation” or “Allogeneic hematopoietic stem cell transplantation” or “HSC” [Title/Abstract].
#5. “Stem cell transplantation” or [MeSH].
#6. #4 or #5
#7. “Chemotherapy” or “High-dose chemotherapy” or “High-dose therapy” [Title/Abstract].
#8. “Chemotherapy” or [MeSH].
#9. #7 or #8
#10. #3 and #6 and #9
#11. Limit #10 to “human”
#12. Limit #11 to “Clinical trial” [Publication Type]
#13. Limit #12 to yr = “-December 2020”

### 4.7 Study selection and data extraction

#### 4.7.1 Study selection and management

**Figure 2:**
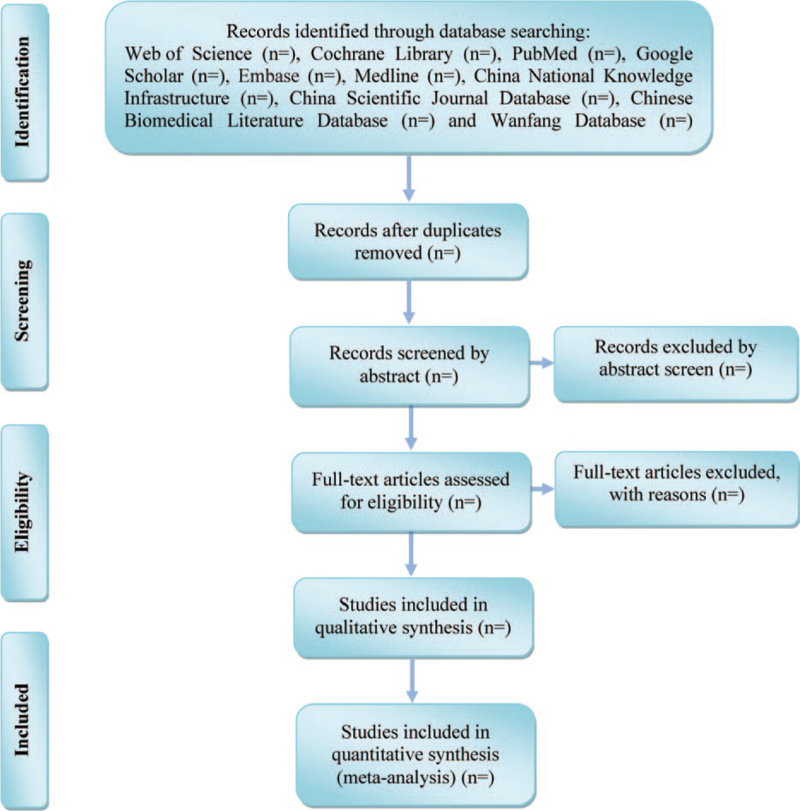
Study selection process for the meta-analysis.

Two experienced authors (Zhang-Shuai Zhao and Wei Shao) will be reviewed independently to identify potential trials by assessing the titles and abstracts. The full text will be further reviewed to determine potential eligible studies. A PRISMA-compliant flow chart (Fig. 2) will be used to describe the selection process of eligible trials. Excluded studies and reasons for exclusion will be recorded. Endnote X7 software will be used for literature managing and records searching. Disagreements between the 2 researchers will be resolved by consensus or by a third independent investigator (Ji-Ke Liu).

#### 4.7.2 Data extraction and management

After screening the text, the 2 investigators (Zhang-Shuai Zhao and Wei Shao) will independently extract the information contained in the eligible literature. The extracted data are as follows:

1. Study characteristics and methodology: country of study, the first author's name, year of publication, randomization, sample size, periods of data collection, follow-up duration, outcome measures, inclusion and exclusion criteria, et al.
2. Participant characteristics: age, gender, stage of disease, diagnostic criteria, et al.
3. Interventions: therapeutic means, autologous or allogeneic HSC, Number of HSC transplants, course of treatment, and duration of treatment, et al.
4. Outcome and other data: tumor response, OS, EFS, QoL, and adverse effects, et al.

### 4.8 Risk of bias assessment

Two researchers (Zhang-Shuai Zhao and Ji-Ke Liu) independently performed assessment of risk of bias in the included RCTs in accordance with the Cochrane Handbook of Systematic Reviewers. The assessment tool includes the following 7 items:

1. random sequence generation,
2. allocation concealment,
3. blinding of participants and personnel,
4. blinding of outcome assessment,
5. incomplete outcome data,
6. selective reporting and
7. other bias.^[28,29]^
8. Each item is divided into 3 levels: low risk, unclear and high risk. The risks of included non-RCTs will be assessed by using Effective Practice and Organization of Care (EPOC) guidelines.^[30]^ Any disagreements will be resolved via discussion with a third researcher (Wei Shao).

### 4.9 Data synthesis

Stata 14.0 (Stata Corp., College Station, TX) and Review Manager 5.3 (Nordic Cochran Centre, Copenhagen, Denmark) statistical software will be used to carry out the data analysis. The risk ratio (RR) was calculated for dichotomous outcomes along with the corresponding 95% confidence interval (CI). Continuous data will be presented as mean difference or standardized mean difference with their 95% CIs. A two-tailed *P* < .05 was considered statistically significant. For survival outcomes, Hazard ratios with corresponding 95% CIs will be extracted from trials or be estimated from Kaplan–Meier survival curves by established methods.^[31]^

## 5 Assessment of heterogeneity

χ^2^ statistics and the *I*^*2*^ statistics will be used to assess the heterogeneity of treatment effects across trials.^[32]^ When the *P* value was >.1, and *I*^*2*^ was <50%, it suggested that there was no statistical heterogeneity and the Mantel-Haenszel fixed-effects model was used for meta-analysis. Otherwise, a random-effects mode will be used to carry out the data analysis.

## 6 Subgroup and meta-regression analysis

When the *P* value was <.1, and *I*^2^ was > 50%. We will explore sources of heterogeneity with respect to age, region and source of HSC by subgroup analysis and meta-regression.

## 7 Sensitivity analysis

Sensitivity analysis of each parameter was carried out by one-by-one elimination method to assess the reliability and robustness of the aggregation results. A summary table will report the results of the sensitivity analyses.

## 8 Publication bias

Funnel plot, Begg and Egger regression test will be performed to analyze the existence of publication bias if 10 or more literatures are included in the meta-analysis.^[33–35]^ If publication bias existed, trim-and-fill method should be applied to adjust the pooled RR.^[36]^

## 9 Assess the quality of evidence

The quality of the evidence will be evaluated by the Grading of Recommendations Assessment, Development, and Evaluation (GRADE) approach,^[37]^ which will be classified into 4 levels (high quality, moderate quality, low quality, and very low quality).

## 10 Patient and public involvement

Not applicable. This protocol of systematic review and meta-analysis does not directly involve patients and the general public. Data will be collected from published articles retrieved from main databases and manual search.

## 11 Dissemination plans

The results of this study will be published in a peer-reviewed journal, and provide more evidence-based guidance in clinical practice.

## 12 Discussion

High dose chemotherapy regimens commonly used to treat refractory neuroblastoma often cause serious adverse effects, which severely affect the hematopoietic system and QoL of patients.^[14,15]^ HSC transplantation can replaces blood-forming stem cells that were destroyed by high-dose chemotherapy.^[19]^ Several studies have reported that autologous or allogeneic HSC transplantation have a unique advantage in the treatment of refractory neuroblastoma by reconstructing the hematopoietic function of patients, and mitigating the progress of the disease.^[20–25,38–47]^ Currently, the largest randomized, phase III trial of autologous HSC for high-risk neuroblastoma was the Children's Cancer Group 3891 study.^[38]^ The study found that EFS among patients with refractory neuroblastoma was significantly better with high-dose chemotherapy and radiotherapy followed by transplantation of purged autologous bone marrow than with chemotherapy alone.^[14,38]^ Autologous HSC are the preferred source for rescue. A possible limitation of using autologous products is the risk of tumor cell contamination in the graft, which has been shown to contribute to relapse.^[39–42]^ Therefore, allogeneic stem cell transplantation has been also tried as salvage treatment in patients with refractory neuroblastoma.^[21,43,44]^ Allogeneic stem cell transplantation would theoretically be preferable in term of relapse-free survival because this has an antitumor effect due to a graft versus tumor effect which is absent in autologous stem cell transplantation.^[39,41]^ Illhardt’ research indicate that haploidentical HSC transplantation is a feasible treatment option that can induce long-term remission in some patients with refractory neuroblastoma with tolerable side effects.^[21]^ Although several recent studies comparing high-dose chemotherapy with HSC transplantation to maintenance chemotherapy have shown improved EFS using this modality,^[20–25,38–47]^ the exact effects of autologous or allogeneic HSC transplantation on tumor response, survival and QoL in patients with refractory neuroblastoma were still not systematically investigated. This meta-analysis will conduct a systematic, comprehensive and objective evaluation of autologous or allogeneic HSC transplantation for refractory neuroblastoma. We hope the findings of this analysis will provide a helpful evidence for clinicians to formulate the best postoperative treatment strategy for patients with refractory neuroblastoma, and also provide scientific clues for researchers in this field.

There are also some possible limitations of our review. First, language bias may exist due to the limitations of English and Chinese studies. Second, there may be some heterogeneity across studies, as the study populations’ baseline for each trial and the study design are difference. When heterogeneity exists, subgroup and meta-regression analysis will be applied to explore the possible sources of heterogeneity.

## Author contributions

**Conceptualization:** Zhang-Shuai Zhao, Wei Shao, Ji-Ke Liu.

**Data curation:** Zhang-Shuai Zhao, Wei Shao.

**Formal analysis:** Zhang-Shuai Zhao, Wei Shao.

**Investigation:** Zhang-Shuai Zhao, Ji-Ke Liu.

**Methodology:** Zhang-Shuai Zhao, Ji-Ke Liu.

**Project administration:** Ji-Ke Liu.

**Resources:** Zhang-Shuai Zhao, Ji-Ke Liu.

**Software:** Zhang-Shuai Zhao, Ji-Ke Liu.

**Supervision:** Wei Shao, Ji-Ke Liu.

**Validation:** Wei Shao, Ji-Ke Liu.

**Visualization:** Wei Shao, Ji-Ke Liu.

**Writing – original draft:** Wei Shao, Ji-Ke Liu.

**Writing – review & editing:** Wei Shao, Ji-Ke Liu.
